# A Perspective on Lifelong Open-Ended Learning Autonomy for Robotics through Cognitive Architectures

**DOI:** 10.3390/s23031611

**Published:** 2023-02-02

**Authors:** Alejandro Romero, Francisco Bellas, Richard J. Duro

**Affiliations:** Integrated Group for Engineering Research, CITIC Research Center, Universidade da Coruña, 15403 Ferrol, Spain

**Keywords:** open-ended learning, lifelong learning, cognitive architectures, autonomous robots

## Abstract

This paper addresses the problem of achieving lifelong open-ended learning autonomy in robotics, and how different cognitive architectures provide functionalities that support it. To this end, we analyze a set of well-known cognitive architectures in the literature considering the different components they address and how they implement them. Among the main functionalities that are taken as relevant for lifelong open-ended learning autonomy are the fact that architectures must contemplate learning, and the availability of contextual memory systems, motivations or attention. Additionally, we try to establish which of them were actually applied to real robot scenarios. It transpires that in their current form, none of them are completely ready to address this challenge, but some of them do provide some indications on the paths to follow in some of the aspects they contemplate. It can be gleaned that for lifelong open-ended learning autonomy, motivational systems that allow finding domain-dependent goals from general internal drives, contextual long-term memory systems that all allow for associative learning and retrieval of knowledge, and robust learning systems would be the main components required. Nevertheless, other components, such as attention mechanisms or representation management systems, would greatly facilitate operation in complex domains.

## 1. Introduction

The standard model of the mind [1] is a picture of the main consensus on the components and structures that should make up a cognitive architecture as well as on their basic relationships. Most recent, and sometimes not so recent, cognitive architectures comply to a certain extent with this model and implement some of the components. However, most of them have been designed with the main objective of studying and/or developing human-like intelligent capabilities and not from an engineering perspective of having robots perform tasks in an ever-more autonomous manner. Clearly, the final objective, i.e., human-like intelligence, is probably the same, but the path towards it is different.

In this paper, we are concerned with the idea of taking robot autonomy to a higher level. This implies providing robots with the capability of handling variability successfully and robustly in the situations/domains they face that were not considered at design time. Variability can be described and addressed at different levels: from slight variations in the operational domains, where it is only necessary to adapt a skill the robot already has; to the designer changing the goal to be achieved in a given domain, in which case the robot must master a new skill; to a more difficult situation in which domains change and the robot must find its own goals and learn to master the skills to achieve them consistently. This last problem is generally called the open-ended learning (OEL) problem, in which a robot must be able to learn to operate in domains that were unknown at design time [2]. We say these robots display OEL autonomy. Additionally, in the most general case, the domains could change continuously and unpredictably, often effectively preventing the robot from being able to master a domain in a single attempt. Therefore, we would be facing a lifelong open-ended learning problem, requiring lifelong open-ended learning autonomy (LOLA).

Increasing the level of autonomy of robotic systems up to LOLA involves jointly solving the OEL [2] and lifelong learning [3] problems. Dealing with these problems goes beyond specific learning algorithms. It requires the ability to manage all the knowledge that is learned so that it can be contextually related and reused, thus facilitating further learning and exploitation. Furthermore, for robots to learn in complex and unfamiliar domains, it is also necessary to manage their motivations, as well as considering other processes, such as attention, representation, and learning. Thus, for robots to autonomously learn to operate in domains that were not considered at design time and build on this knowledge to address new domains as they emerge during their lifetime, the capabilities mentioned above (and probably some more) must be integrated and regulated. This is the job of cognitive architectures, and these have been studied for decades [4]. However, as we have already mentioned, the purposes for which each one was created were different and usually not related directly to LOLA. Consequently, it makes sense to carry out a brief overview of the main cognitive architectures found in the literature and characterize them in terms of the level of autonomy they allow and their possible adequation to LOLA. Thus, the objective of this paper is to provide an overview of the main types of cognitive architectures that have been developed in recent decades and select some examples of each, in order to characterize them in terms of LOLA-related capabilities. Thus, researchers in the field of autonomous robotics will have an updated and reliable reference of the state of the art in this area.

To this end, Section 2 provides a general classification of cognitive architectures based on the work carried out in [4] and establishes a series of requirements for LOLA. In this section, a series of representative and well-known architectures are selected, and their main sub-systems and components are evaluated. Section 3 is devoted to a discussion of how these architectures fulfil LOLA capabilities, pointing towards their strengths and, more importantly, what is lacking. Finally, Section 4 provides a series of conclusions and paths for future developments to create a new generation of LOLA-capable cognitive architectures.

## 2. Cognitive Architectures and LOLA

Cognitive architectures are structures that artificially implement cognition [5]. They allow learning, storing, using, and reusing knowledge and can also contemplate developmental or other integration strategies to produce new higher-level knowledge nuggets from the elements stored in their memory. Many types of cognitive architectures have been developed over the past four decades, each one addressing different aspects of cognition. They generally base their operation on their abilities to interpret, index, and sort the different knowledge elements they require based on their content. However, they use different approaches to this end. Following [4], cognitive architectures can be firstly classified into three basic groups according to the type of representations they can manipulate:

- Symbolic: Most of the traditional cognitive architectures, especially in their initial form, belong to this group, although some of them have been later hybridized. They are characterized by representing concepts through symbols and having predefined instruction sets to manipulate them. This makes them excellent systems in terms of planning and reasoning. However, for the same reason, they present grounding problems and lack the robustness and flexibility needed to adapt to the changing conditions of real environments. In addition, the designer assumes a high degree of knowledge about the domains and tasks to be performed and, therefore, provides a lot of knowledge in the form of specific representations or even complete sets of rules in some cases. They are, therefore, limited to use in an abstract framework and are not generally ready to tackle the LOLA problem. We can take as representative examples of this group ACT-R [6], CLARION [7], 4CAPS [8], or SOAR [9]. SOAR, developed by Allen Newell, Paul Rosenbloom, and John Laird, is one of the most-studied architectures. It was created in 1982 but it has undergone different improvements and additions, including hybridizations, throughout the years. CLARION, 4CAPS, and ACT-R were also built as symbolic architectures but, over the years, have also been hybridized. Their main goal initially was to study and emulate human cognitive processes, and, thus, they led to very few real robot applications. Along the same line, we can also find EPIC [10], created in 1980, whose main objective was to replicate the human motor system. On the other hand, an example of architecture of this group created to be used in robotics is ICARUS [11]; however, it has been tested mainly in experiments related to solving puzzles and driving games.
- Emergent: Based on sub-symbolic or connectionist approaches, they often follow developmental principles [12,13] that seek to progressively build system knowledge from scratch through direct interaction with the world. In them, knowledge is often represented and distributed through neural networks. This approach provides a direct path to the autonomous construction of high-level knowledge, avoiding grounding problems. Thus, they aim to solve the problems of adaptation to the environment and learning through the concatenation of multiple models in parallel, where information flows through activation signals. However, this introduces a high level of complexity in development and the need for very long learning and interaction processes, which is, of course, very costly when considering robotics applications. It also causes the system to lose transparency, as knowledge is no longer represented by well-understood symbols and rules, instead being distributed throughout the network. Some examples of these architectures are MDB [14], GRAIL [15], or SASE [13]. In the case of SASE, its main purpose is the autonomous learning of models. GRAIL (and its modified versions M-GRAIL [16], C-GRAIL [17], and H-GRAIL [18]) comes from the field of intrinsically motivated open-ended learning (IMOL), and its focus is on the handling of motivations to seek and relate goals and skills. MDB is a long-standing project started at the end of the 1990s that seeks to implement an evolutionary cognitive architecture suitable for developmental processes in a direct bottom-up approach so that knowledge is always grounded. Finally, we can also include MicroPSI [19] in this group, which was developed in 2003 and combines associative learning, reinforcement learning, and planning in order to allow autonomous systems to acquire knowledge about their environment.
- Hybrid: Finally, the group of hybrid architectures consists of those that use symbolic representations at higher processing levels but include emerging connectionist paradigm-like sub-symbolic representations at the low level. These approaches have become quite popular for addressing low-level grounding and domain adaptation problems, but they still require many adjustments to construct symbolic information. In fact, even though there are researchers trying to provide autonomous approaches to bridge the gap between sub-symbolic and symbolic representations [20], they are still not very common in cognitive architectures. This makes these architectures difficult to adapt to general use cases in robotics, and their implementations tend to focus on specific functionalities. Recent examples of such architectures (apart from the previously mentioned symbolic architectures that have been hybridized) are, on the one hand OpenCogPrime [21], which is a product of the ideas from the artificial general intelligence (AGI) community, which seeks to address intelligence through a holistic approach and not by creating specific AI-based modules that are then integrated. On the other, we have MLECOG [22], which was created by Janusz A. Starzyk and James Graham in 2017 with the aim of moving towards greater autonomy by including motivations and goal creation. In this group, we also find architectures such as ADAPT [23], designed to solve computer vision problems, or LIDA [24] and DUAL [25], which were both designed to study human cognitive processes. A set of additional architectures emerged from European research projects. Representative examples of these are IMPACT [26], developed by the same authors as GRAIL, which combines planning and reinforcement learning algorithms with intrinsic motivations to represent autonomously learned skills, or iCub [27], created to control the robot to which it gives its name and aimed at the study of how newborns learn.

In addition to the type of representation, we are going to consider additional features to classify the existing cognitive architectures here. From the perspective of lifelong open-ended learning autonomy, and from a functional viewpoint, we must bear in mind that architectures should contemplate, at least, the following components:

- A motivational system that enables open-ended learning, i.e., that allows the robot to discover new goals and select which ones are active at each moment in time.
- A memory system that permits storing the acquired knowledge and relating it contextually, that is, without having to externally label the knowledge, to facilitate its reuse in the right conditions so that lifelong learning is made possible.
- An online learning system that facilitates acquiring knowledge about the different goals discovered in the different domains, as well as about how to achieve them during robot operation (skills).
- Some type of attention system that helps to reduce the sensory and processing load of the system when operating in real-world conditions would also be very convenient.

We have selected a set of fifteen well-known cognitive architectures representative of the three architecture types and have analyzed their structure and components according to the previous four features. The criteria for selecting these architectures were that their development is still ongoing and that they have practical applications. For compactness, Table 1 provides a summary of their characteristics and the requirements they meet with regard to their LOLA capabilities. In the following subsections, we describe how they address the four main components mentioned above in an individual manner.

### 2.1. Learning

Starting with the learning component, and even though it may seem rather obvious, it is important to note that possessing the ability to learn is fundamental to be able to address LOLA. However, not all cognitive architectures presented in the literature show this capability. In fact, many symbolic architectures do not implement learning mechanisms and, therefore, their knowledge must be introduced by the designer when they are built. This implies that the domains in which the robot will operate must be known at design time, contradicting the open-ended learning principle. Examples of these are EPIC [10] or 4CAPS [8]. In addition, others within the symbolic or hybrid group, such as DUAL [25], ADAPT [23], and ICARUS [11], incorporate learning capabilities, but mostly through top-level rule modification, without a versatile and unrestricted ability to create new rules for new domains. Only within the group of emerging cognitive architectures, such as iCub [27] or MDB [14], and in a small group of hybrid architectures, such as MicroPSI [19], can versatile low-level learning mechanisms be found. Consequently, only these types of architectures would be candidates for achieving LOLA in robots from the point of view of learning.

Another required property of the learning systems in LOLA is supporting online operation. Lifelong learning requires model creation and transferring learning from previously acquired knowledge as a core feature. In this sense, the number of existing cognitive architectures that perform online learning is scarce, and even more so if we look for reliable solutions that have been tested in real operation. One of the exceptions is the MDB [14], which contains an online learning procedure based on neuroevolution [29,30] and an episodic memory management method that has been validated in simple real robot experiments.

### 2.2. Motivational System

On the other hand, when we talk about a cognitive architecture having a motivational system, we refer to the fact that it should be endowed with a mechanism in charge of determining what the robot should strive for in a given domain at each moment in time. This mechanism can have different functionalities, from being able to guide the robot towards the achievement of a goal, to allowing for the selection of which goal/goals are active at each moment in time, or even being able to guide the robot to discover new goals. It is worth remembering that possessing these three qualities is what may allow the robot to be able to perform OEL. Considering these qualities, a classification of motivational systems into different levels can be established. The levels we consider in this paper are the following:

- Level 0: The robot has a specific goal set by the designer and the motivational system is able to guide the robot towards the achievement of that goal.
- Level 1: The robot has a series of goals set in advance by the designer and the motivational system is able to select which goals should be active at any given moment in time and guide the robot towards their achievement.
- Level 2: The robot has a series of goals set in advance by the designer and the motivational system is able to select which goals are active at any given moment in time and is capable of autonomously generating sub-goals to reach those goals.
- Level 3: The goals/domains are not known at the time of design and the motivational system is able to discover goals, select which ones are active, and guide the robot towards their achievement.

It is important to note that levels 0, 1, and 2 imply that the designer knows in advance the goals and domains in which the robot will operate, while level 3 is domain-independent and will be the one required to provide robots with OEL autonomy.

At level 0, we find all the architectures that do not have an explicit motivational system. They allow the robots/agents they control to achieve the specific objective for which they are designed. Examples are symbolic architectures such as EPIC [10], hybrid architectures such as ADAPT [23] and DUAL [25], or emergent architectures such as SASE [13].

If we go to level 1, we have examples such as CLARION [7], which uses a motivational system based on drives. These drives have goals associated with them beforehand, so that the activations of the goals depend on the value of the drives. In other architectures such as LIDA [24], the motivations of the system are set in the form of artificial sensations and emotions. This allows it to appropriately select its goals and, consequently, the actions with which to act on the environment. Something similar happens in OpenCogPrime [21], where human motivations of feelings and beliefs are modeled through a motivational system based on the concepts of magicians and anti-magicians.

At level 2, we find multiple different implementations of motivational systems. Architectures such as SOAR have a motivational system that allows them to generate their own subgoals from goals predefined by the designer [9] as a previous step to be able to address a problem. MDB [14] also allows the intrinsic change of goals or motivations, and the generation of subgoals by introducing a satisfaction model. In MDB, the degree of fulfillment of motivations is based on both internal and external perceptions of the agent. This is similar to how MLECOG [22] handles motivations and their action choices based on pain/need (and other factors such as distance and availability). Moreover, in MLECOG, only a few motivations are given to the system, with all others being developed internally. On the other hand, MicroPSI [19] also has a motivational system based on needs and drives, so that MicroPSI agents use pleasure/distraction signals related to the satisfaction of those drives. Finally, in the iCub [27] architecture, it is the affective state that is in charge of providing the motivational cues. Thus, it has affective factors (motivations) that allow it to acquire knowledge and validate it.

Finally, only two of the architectures found in the literature present a motivational system that could be suitable for carrying out OEL. GRAIL [15] and IMPACT [26] present motivational systems composed of intrinsic motivations based on competence. These systems allow them to autonomously learn new skills based on the self-generation of goals driven by intrinsic motivations (intrinsic goals).

### 2.3. Decision Systems and Contextual Memory

The final purpose of a cognitive architecture is to decide on the actions to be executed. The decision processes used for deciding on actions almost always revolve around two main concepts: prospection and experience. Prospection is related to the anticipation or prediction of future states (really beliefs in the context of cognitive architectures) so that they can be evaluated using a motivational system to allow for the selection from the potential actions or policies as a function of the expected achievement of its goals [31,32]. This deliberative process requires performing predictions into the future, usually carried out by models (world models, internal models), and evaluations of the predicted beliefs (points in belief space) by means of utility functions. Of course, the memory-related problem here becomes how to find the appropriate models and/or utility functions in order to perform deliberation in the current situation.

On the other hand, experience is related to direct or statistical associations or relationships the system has found among its knowledge components or knowledge nuggets (models, policies, perceptual classes, etc.) when it was successful at achieving a goal (or, in some cases, even unsuccessful). These relationships allow the system to directly choose an action or policy without any prospection or evaluation if it can determine the context it is in, that is, if it can determine in which world it is operating, what its goal is, and what its current perception is. Through a structure of previously observed relationships, when a known context arises, it can directly activate the action or policy that produced a successful result in a previous instance of the same or a similar context. In the terminology of many authors, the decision process has been automated, as it does not require any prospection for its completion [33]. This is the idea of associative learning as the learning process by means of which an association is established between two or more stimuli or a behavior and some stimuli. The key here is to progressively create and associate different knowledge nuggets within a long-term memory (LTM) in a meaningful and general manner, that is, to provide compact experiential representations so that hypotheses can be made on the actions to take when faced with similar perceptions in different contexts.

This makes LTM critical for addressing cognition [34]. However, probably due to the fact that humans are not conscious of the contents of LTM except when they are brought into working memory, its critical role in cognitive activity is often ignored. This has led to most authors creating artificial cognitive architectures paying very little attention to this system except as a passive storage container for knowledge. A computer architecture-like analogy of the mind has been the predominant paradigm: memory as a hard disk with discrete encoding, storage, and retrieval functions.

More recently, authors such as Wood [34] or Fuster [35] state that to achieve properties that are necessary for autonomy, e.g., adaptability, flexibility and robustness, LTM must be situated within the perception–action cycle of adaptive behavior and must operate in an associative and distributed manner. They argue that some of the most relevant mechanisms for lifelong cognition are those related to an associative LTM and its operation.

Therefore, in order to achieve LOLA, where most of the knowledge elements are acquired by the autonomous system itself and, thus, cannot be externally labeled, it seems that there is quite a strong need to establish a memory structure that can operate as a dynamic associative component to support the different decision processes required.

If we look at the type of memory systems presented by the different architectures in the light of the previous comments, we can distinguish two main groups. On the one hand, we have a series of architectures that have a more classical computer-type memory. In them, all the knowledge generated is stored under a label. In this group, we can find architectures such as EPIC [16], 4CAPS [7], or SASE [11]. On the other hand, we have a series of architectures that present an associative memory system, more similar to natural memories. These associative memories are characterized by the fact that they are able to relate knowledge through the context in which it can be used. Thus, as discussed, they would be the most appropriate to be able to achieve LOLA. This group includes architectures such as ACT-R [15], MLECOG [14], OpenCogPrime [13], or CLARION [6]. However, in these architectures, the contextual associations are implemented by hand by the designer and are not created autonomously, thus defeating the purpose of LOLA. Therefore, it is necessary to address the problem of establishing contextual or associative memories that are filled in by the cognitive architecture itself by including mechanisms that allow determining when a context is relevant in order to be stored as such in the LTM, as well as mechanisms for the contextual retrieval of knowledge. This second aspect has already been partially addressed in the construction of architectures such as ACT-R [15], MLECOG [14], and OpenCogPrime [13]. However, the first one is still an open problem in terms of its inclusion in general-purpose cognitive architectures.

### 2.4. Attention

Finally, another component present in most of the reviewed architectures, and that could help to cope with LOLA, is attention mechanisms. Attention is necessary to reduce the amount of sensory information for real-time operation and select the sensory information most relevant to the current situation. Attention allows the architecture to manage real-time operation, reducing the amount of information processed and, consequently, the reaction time of the system. Examples of architectures with attention mechanisms are MicroPSI [19], LIDA [24], MLECOG [22], or iCub [27].

## 3. Discussion

Most of the architectures shown in Table 1 were not created with the objective of achieving higher levels of autonomy in real robots, but with the objective of demonstrating/imitating human behaviors. Moreover, most of the existing architectures were designed for intelligent agents, and not for real robots. Therefore, they are not really prepared to work in real environments and manage continuous perceptual spaces. Only some emergent architectures have been tested using real robots in laboratory experiments to verify specific cognitive functionalities [27]. Additionally, the fact that none of the existing cognitive architectures have been explicitly designed to address the LOLA problem implies that most lack some of the necessary components/functionalities to be able to achieve it. Table 2 shows a summary of the four features commented on above and the architectures that include them in green. As can be observed, there is no existing approach that covers all of them (the whole column in green). Many implement motivational systems, although most of them are not prepared to deal with OEL and remain at lower levels of autonomy. Additionally, some of the architectures include attention systems and have low-level learning mechanisms. However, very few of them include an associative memory capable of handling contexts, and these are usually constructed by the designer.

Hence, although there are no architectures explicitly designed to address the problem of achieving LOLA in a general way, there is quite a lot of work on different aspects of this field. Thus, there are examples in the literature of architectures such as GRAIL [15] or IMPACT [26] that have been tested in different OEL problems. However, they are run in simulations [26] or they only address a specific part of the robotic system and, therefore, cannot be translated to reality [15]. Other architectures, such as ACT-R, have addressed knowledge reuse problems [36], although without using real robots, and starting from knowledge previously introduced by the designer.

It must be pointed out that some of the four features established in Section 2 have been addressed to a greater or lesser extent in specific fields. In this line, the intrinsically motivated open-ended learning (IMOL) framework has made great contributions towards achieving agents capable of operating in an open-ended manner and autonomously acquiring knowledge and skills to solve tasks that are not known at design time. These approaches have been used in a wide variety of applications such as state-space exploration [37,38,39], knowledge gathering [40,41], autonomous skill learning [18,42,43,44] or autonomous goal selection [15,18]. However, despite these advances, IMOL systems are still difficult to use in real-world applications. This is because these systems are designed to acquire the maximum possible knowledge from the interaction of the robot with the environment, but without considering the purpose for which the robot was designed. This results in an unbounded and unfocused learning that is not adapted to the specific needs of a service robot. A solution to this problem could consist of providing a motivational mechanism capable of considering and balancing different typologies of motivations. However, this topic is still under study [45]. Moreover, another problem that is not yet solved is the design of specific motivations to trigger representation/redescription processes. It is important to look for motivational mechanisms that allow for seeking better representations when necessary, since this is something that, as has been commented on before, is critical for facilitating learning and, more importantly, abstraction.

Regarding lifelong learning, fields such as transfer learning [46] or continual learning [47] present very promising approaches to the problems of knowledge reuse and task learning in multiple domains, respectively. These approaches have proven to be effective for deep learning or supervised learning. However, they are not yet applicable to real robotic problems, since the former are not able to solve the issue of catastrophic forgetting [48,49], while, in the latter, the tasks to be performed and the domains of operation of the robot must be known in advance by the designer. Thus, as they are not intended for LOLA problems, they do not fully cover the needs that arise in this field.

Finally, it is interesting to comment that all the architectures have implicitly or explicitly assumed that robot cognitive systems are given specific and appropriate state-space representations by their designers. That is, designers decide what is relevant from the robot’s sensory flow and how these relevant features are represented. Consequently, the learning mechanisms for architectures have focused on how to learn whatever knowledge components the architectures require (direct or inverse state transition models, utility models, policies, etc.) using these predefined state-space representations. Therefore, it seems that it would also be important to start addressing the issue of learning representations within the framework of cognitive architectures in order to provide paths for the simplification of the learning processes as well as for the introduction of abstraction capabilities.

## 4. Conclusions and Perspective

Most current applications of autonomous robots consider a very limited range of autonomy, usually dealing with a limited number of unexpected disturbances in the domain the robot is designed for. They seldom face the problem of autonomously setting goals in previously unknown domains (open-ended learning autonomy) nor, consequently, using experience from previous domains to facilitate current learning (lifelong open-ended learning autonomy, LOLA). Cognition and cognitive architectures have been purported as a way to address problems that require higher levels of autonomy. However, the mostly programmed-in symbolic representations of traditional general-purpose cognitive architectures are not up to the task due to their grounding and domain adaptation problems. Hybrid approaches, on the other hand, have become quite popular to address grounding and domain adaptation at a low level, but they require a lot of tweaking of the symbolic information in the higher levels, thus generally making them inadequate for open-ended learning situations. Finally, most emergent cognitive approaches have never been completely integrated into full cognitive architectures or tested on real market use cases. In fact, most developments are incomplete and only address a specific part of the robotic system and, thus, require more work to be ported to reality.

In this work, we identify four basic components required for cognitive architectures that support LOLA: a motivational system, a contextual memory system, an online learning system and, finally, an attention system. In general, there has been a lot of work on several aspects pertaining to LOLA, but mainly within areas outside the cognitive architecture realm and hardly ever considering these four components together. These works range from intrinsically motivated structures to provide for goal discovery, to different approaches to knowledge reuse. Thus, the open question that needs to be addressed now is how to integrate this work within operational cognitive architectures that provide the four structural components and the internal operational mechanisms needed to achieve the LOLA objective in a way that does not constrain their performance and possibilities.

**Figure 1:**
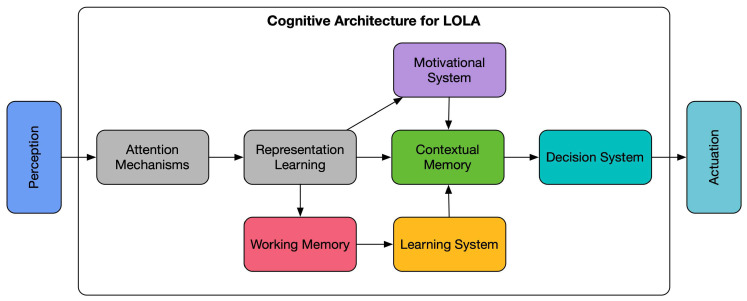
Components that a cognitive architecture should contemplate to be able to reach the autonomy and learning capacity necessary to achieve LOLA.

This opens up a whole set of research paths towards constructing a cognitive architecture that is able to support and relate the knowledge designed by the robot’s creator with the knowledge discovered and learned by the robot itself, in such a way that useful decisions can be made. Such a structure must be able to adapt its decision-making processes to its level of knowledge. Therefore, it is important that it can balance and complement deliberative and reactive decisions. The latter because they are faster and more efficient, while the former are the ones that will allow the robot to explore the different domains, discover new goals in them, and acquire knowledge on how to reach them. Therefore, after the review presented in this article, we have found that this architecture must contemplate, among others, the components shown in the schematic of Figure 1 to provide it with the aforementioned autonomy and lifelong learning capacity. Additionally, and with the objective of making the operation of the architecture more efficient, the inclusion of self-maintenance and autonomous internal knowledge enhancement procedures should also be contemplated. Similarly, mechanisms to obtain a balanced integration of deliberative and reactive decision-making processes will also be important.

## Figures and Tables

**Table 1:** Features of the cognitive architectures under study.

Architecture	Type (Following [4])	Design Objective	Motivational System Level	Learning System	Contextual Memory	Attention Mechanism	Real Robot Applications
EPIC [10]	Symbolic	Emulate human cognition	0	NO	NO	YES	NO
ICARUS [11]	Symbolic	Robotics	1	YES (rule-based)	NO	NO	NO
ADAPT [23]	Hybrid	Computer vision	0	YES (rule-based)	NO	YES	NO
CLARION [7]	Hybrid	Emulate human cognition	1	YES	YES	NO	NO
LIDA [24]	Hybrid	Emulate human cognition	1	YES	YES	YES	NO
iCub [27]	Hybrid	Robotics	3	YES	NO	YES	YES
SOAR [9]	Hybrid	Robotics	2	YES	YES	NO	YES
OpenCogPrime [21]	Hybrid	Artificial General Intelligence	1	YES	YES	NO	NO
DUAL [25]	Hybrid	Emulate human cognition	0	YES (rule-based)	YES	NO	YES
4CAPS [8]	Hybrid	Emulate human cognition	0	NO	NO	NO	NO
ACT-R [28]	Hybrid	Emulate human cognition	0	YES	YES	YES	YES
MLECOG [22]	Hybrid	Autonomy	2	YES	YES	YES	NO
IMPACT [26]	Hybrid	Robotics	3	YES	NO	NO	NO
MicroPSI [19]	Emergent	Autonomy	2	YES	NO	YES	NO
GRAIL [15]	Emergent	Robotics	3	YES	NO	NO	YES
MDB [14]	Emergent	Robotics	2	YES	NO	NO	YES
SASE [13]	Emergent	Model learning	0	YES	NO	NO	YES

**Table 2:** Main components to achieve LOLA and cognitive architectures that implement them.

Architecture	EPIC	ICARUS	ADAPT	CLARION	LIDA	iCub	SOAR	OpenCogPrime	DUAL	4CAPS	ACT-R	MLECOG	IMPACT	MicroPSI	GRAIL	MDB	SASE
**Motivational system for OEL**	*0*	*1*	*0*	*1*	*1*	*3*	*2*	*1*	*0*	*0*	*0*	*2*	*3*	*2*	*3*	*2*	*0*
**Learning system**		rule-based	rule-based						rule-based								
**Contextual memory**																	
**Attention mechanism**																	

## Data Availability

No new data were created or analyzed in this study. Data sharing is not applicable to this article.
